# From board to bedside – training the communication competences of medical students with role plays

**DOI:** 10.1186/1472-6920-14-135

**Published:** 2014-07-05

**Authors:** Katharina Luttenberger, Elmar Graessel, Cosima Simon, Carolin Donath

**Affiliations:** 1 Division of Medical Psychology and Medical Sociology, Department of Psychiatry and Psychotherapy, Friedrich-Alexander-Universitaet Erlangen-Nuremberg, Erlangen, Germany

**Keywords:** Teaching Materials, Teaching, Problem-based Learning, Education, Students, Medical, Students, Health Occupations, Schools, Medical, Academic Medical Centers, Psychology, Medical

## Abstract

**Background:**

Role plays and standardized patients are often used in medical education and have proven to be effective tools for enhancing the communication skills of medical students. Most course concepts need additional time and teaching staff, and there are only a few studies about role plays in the preclinical segment.

**Methods:**

We developed a highly consolidated concept for the curricular course of 2^nd^-year medical students, including ten role plays about five subjects: anamnesis, shared decision making, prevention, breaking bad news, and so-called “difficult interactions”. Before the course, all students were asked about their expectations and attitudes toward the course. After the course, all students rated the course, their individual learning progress, whether their expectations had been fulfilled, and re-evaluated their attitudes. Questionnaires were self-report measures and had a quantitative and a short qualitative section and were analyzed with descriptive statistics. Group differences (sex, age, role played) were evaluated with *t* tests at a Bonferonni-corrected significance level of p = .03 and the non-parametric U-tests.

**Results:**

Implementing this practical course concept is possible without incurring additional costs. This paper not only shows how that can be done but also provides 5 examples of role scripts for different training subjects. The course concept was highly appreciated by the students. More than 75% felt that they had learned important communication techniques and would be better able to handle difficult situations. Playing the doctor’s role was felt to be more useful than playing the patient’s role. Women admitted a higher degree of shyness in the beginning and gave higher ratings to their learning progress than men. Students’ most frequent wish in the qualitative analysis was to be able to play the doctor’s role at least once. The students’ answers showed a differentiated pattern, thus suggesting that the influence of social desirability was minimal.

**Conclusions:**

Practical skills can be taught successfully in the preclinical stage of medical education even without an increase in resources. The course concept described in this article provides an effective means by which to do so.

## Background

There is a great deal of evidence indicating that the quality of communication between physician and patient influences the patient’s health outcome [1-4]. Doctors’ levels of work stress, quality of diagnosis [2], and even malpractice claims are associated with their communication skills [5]. Nevertheless, in recent times, more and more critical comments about the quality of physician-patient encounters have arisen. A popular German magazine, for example, ran cover stories on physicians entitled “Speechless in consultation hours” [6] and “Physicians’ cardinal errors” [7]. A recent study on whether patients with chronic bronchial asthma adhered to treatment also showed that adherence was significantly related to the quality of the information given to the patient. Nevertheless, the authors showed that in patients’ daily routines, only about 55% indicated feeling “totally sufficiently” informed about their medications’ mechanism of action [8]. Effective communication training methods for doctors-to-be are therefore needed. Maguire and Pitcathy claimed in their recent clinical review that effective methods of communication skills training require the opportunity to practice “under safe conditions” and to receive feedback [2]. To date, it seems clear that teaching methods that offer students practical experience have greater benefits than pure lectures [9-12]. In recent years, many universities have therefore introduced practice-related courses that use either role plays or standardized patients (SPs). Against previous assumptions, in a 2007 review [13] and several more recent randomized controlled trials (RCT) [14-17], the use of peer role playing has been shown to be as effective as the use of standardized patients. In addition, the use of peer role playing has appeared to create an appreciation for empathy in future doctors as they are given the opportunity to stand in the shoes of their future patients [14,15,18]. Furthermore, it is less time consuming and less expensive than the use of standardized patients. The greater amount of “realism” brought by SPs (people who are unknown to the students, of different ages, etc.) therefore does not seem to compensate for the handicaps of using these actors as they have been found to behave significantly differently from “real patients” [19]. In Germany, only a few universities have introduced role-play training into the compulsory curriculum of their preclinical medical students in the first preclinical part of their studies (“Vorklinik”) (for example: [20]). Most publications refer to communication skills programs that are offered as add-ons to the medical curriculum or to classes for more advanced students. Such programs train not only communication but also technical competences (for example: [21]). Although the effectiveness of simulation-based teaching methods has already been widely demonstrated, the important question remains how to implement them in a cost-efficient way [22]. We wanted to offer practical communication training to our preclinical students so that they could focus on aspects of communication that would prepare them for their first encounters with real patients. Thus, the main focus of this paper is to show how such a training concept can be implemented without additional financial or human resources so that it can potentially be used at any university regardless of economic resources. Therefore, we designed a training concept for 2^nd^-year medical students in Erlangen as a mandatory course in which every student takes an active part in at least one role play and witnesses 9 others. The course concept is presented in the Methods section, and the methods of implementation are presented in the Results section. The class is accompanied by a portfolio in which the students document two interviews that they conduct on their own with real patients, including the students’ reflections about their communication skills and improvements. The portfolio concept has been published elsewhere [23]. The communication course was evaluated with regard to the acceptance of the course concept, learning goals, expectations, and gained competences by 182 medical students in the years 2012 and 2013 (winter semester). A review of the literature had indicated gender differences [21] and different impacts of the type of role played [15]. Therefore, we tested for differences in these groups and – as there were not many studies on medical students in their 2^nd^ year – we also tested for differences according to the age of the students in the analyses. The presentation of these results was the second goal of this paper.

## Methods

### Communication training: course concept

Second-year medical students participated in a mandatory training course “Physician-patient communication” for 12 hours in groups of about 15 persons. The course was held across 6 days for 2 hours on each day, the minimum amount of time that German medical students are required to spend on the subjects of Medical Psychology and Medical Sociology in their 3^rd^ semester. In the first class, a short introduction to the concept of the course was given, followed by a review of communication theories, which students had already learned about in their first year.

In the next five lessons, the students performed/witnessed a total of 10 role plays covering five different subjects in each course: anamnesis, shared decision making, prevention/motivation, breaking bad news, and “difficult” interactions. The theoretical framework behind all of these situations was presented in a lecture format during the first semester. For anamnesis, we used widely accepted models on how to structure anamnesis [24] and general concepts of professional encounters derived mainly from Rogers’ client centered therapy [25]. Shared decision making and prevention/motivation were founded on the concept of motivational interviewing [26]. The basic principles were also introduced during the first semester and repeated in the first session, although we did not go into too much detail on the theory and rather focused on the feedback. Breaking bad news was based on the SPIKES Model [27], which was discussed in detail during the first semester. In the “difficult-interaction” class, students chose two of three role scripts, which focused on different problems in clinical life consisting of patients with a great fear of side-effects, with somatic symptom disorder, and without a clear diagnosis. Here, counseling techniques were applied in accordance with the specific situation. Examples of one role script for each session are provided in the Additional file 1. Students were allowed to choose the physician’s or the patient’s role. The professor decided which of the five subjects students would perform according to the role sets (for example, a female student always played the role of a pregnant patient, and so on). If there were fewer course participants than role players needed, volunteers were encouraged to play a second role.

All physician-players received their role set in the first lesson. The case descriptions for “physicians” included all necessary medical information to allow the students to concentrate on communication. “Patients” received their case descriptions at the beginning of the respective lesson.

Every “playing lesson” began with the patient-player receiving his or her role description and preparing outside the classroom. During that time, the physician-player reported all she or he already knew about the case to the other students. Questions about the case or about possible communication problems could be discussed, and the purpose of the encounter was defined. Then the patient-player had the opportunity to discuss potential problems with the professor. Afterwards, the patient-player came into the classroom again, and the role play began. The role play lasted for approximately 10 to 20 minutes. The other students were asked to document the encounter on a feedback form that offered subject-related questions. The feedback form always contained one section in which everybody could write freely what they liked/disliked about the role play. After every session, the following questions were asked: “Which interaction model did you see represented by the doctor’s behavior?” and “Did the doctor make an empathetic statement? Please write it down verbatim”. The further questions were directly related to the subject; for example, for the “Breaking bad news” lesson, the different parts of the SPIKES Model were presented along with the request to state how the doctor had performed each part.

After the role play, both players were asked to share their experiences and define important subjects or questions for feedback. Then the observers’ feedback was given in a structured way: First, positive feedback was given, then problems were discussed. The professor moderated the discussion and wrote the important comments as “dos and don’ts” on the board.

### Methods of evaluation

#### Design

In the winter semester 2012/2013, N = 182 medical students in their third semester of medical studies evaluated the course “Training patient interviews using role plays” at the beginning and at the end of their six-session course. The questionnaires were applied as part of their teaching evaluation, constituting a standard procedure at the University of Erlangen-Nuremberg for reasons of quality assurance. Therefore, the evaluation was approved by the Friedrich-Alexander-University of Erlangen-Nuremberg. It was voluntary and anonymous, and students could refuse to participate without incurring any kind of penalty or differential treatment. Data sheets were stored in a locked cabinet. The procedure complies with the declaration of Helsinki. The qualitative part of the study adheres to the qualitative research review guidelines (RATS- guidelines).

#### Sample

The sample consisted of 182 students in the preclinical part of their medical studies, this were all students participating in the course. The mean age of the students was 22.0 years (SD = 3.8), and the majority of them were female (59.9%). Half of them (50.3%) played the role of the doctor at least once; the rest of the students played the patient role (49.7%). After the course, 173 of them (95.1%) completed the questionnaires.

#### Instruments

At the beginning of the first class, the students were given a questionnaire containing ten questions that had to be answered on a five-point scale (Likert-type) with the categories “agree completely”, “agree in part”, “so/so”, “hardly agree”, and “do not agree at all”. The questionnaire collected only quantitative data. In the last lesson of the course, a questionnaire with 14 questions was distributed (see above for answer categories) for further quantitative assessment. In addition, the students were asked two qualitative questions: “What did you like about the course?” and “What should be changed and how?” The final two questions were voluntary. The formulation of the quantitative items can be found in Tables 1 and 2.

**Table 1:** Descriptive statistics: beginning of course (n = 182 completed questionnaires)

**No./statement**	**Response category**				
	**Agree completely**	**Agree in part**	**so/so**	**Hardly agree**	**Do not agree at all**
	**n (%)**	**n (%)**	**n (%)**	**n (%)**	**n (%)**
	**(1)**	**(2)**	**(3)**	**(4)**	**(5)**
1) A course on doctor-patient communication is a good idea in general.	127	47	8	0	0
	(69.8%)	(25.8%)	(4.4%)	(0.0%)	(0.0%)
2) I like that the contents of the course are not taught only in a lecture format.	77	70	30	4	1
	(42.3%)	(38.5%)	(16.5%)	(2.2%)	(0.5%)
3) I think it is important to play the role of the doctor at least once during the course.	76	68	27	10	1
	(41.8%)	(37.4%)	(14.8%)	(5.5%)	(0.5%)
4) I think it is important to play the role of the patient at least once during the course.	54	59	46	19	4
	(29.7%)	(32.4%)	(25.3%)	(10.4%)	(2.2%)
5) I would like to play more than one role.	11	21	54	65	31
	(6.0%)	(11.5%)	(29.7%)	(35.7%)	(17.0%)
6) When I imagine conducting an interview in front of the class, I get nervous.	21	46	50	43	22
	(11.5%)	(25.3%)	(27.5%)	(23.6%)	(12.1%)
7) Concerning the feedback I will receive after playing a role, it is important to me that I be evaluated fairly.	171	9	2	0	0
	(94.0%)	(4.9%)	(1.1%)	(0.0%)	(0.0%)
8) I have already independently conducted patient interviews before (e.g. as a nurse).	80	36	22	15	29
	(44.0%)	(19.8%)	(12.1%)	(8.2%)	(15.9%)
9) I would like to learn how to manage difficult communication situations with patients.	136	39	5	2	0
	(74.7%)	(21.4%)	(2.7%)	(1.1%)	(0.0%)
10) I think I will learn something useful for my later job as a doctor.	39	101	39	3	0
	(21.4%)	(55.5%)	(21.4%)	(1.6%)	(0.0%)

**Table 2:** Descriptive statistics: end of the course (n = 173 completed questionnaires)

**No./statement**	**Response category**				
	**Agree completely**	**Agree in part**	**so/so**	**Hardly agree**	**Do not agree at all**
	**n (%)**	**n (%)**	**n (%)**	**n (%)**	**n (%)**
	**(1)**	**(2)**	**(3)**	**(4)**	**(5)**
1) I was satisfied with the way the instructor taught the class.	83	70	17	3	0
	(48,0%)	(40.5%)	(9.8%)	(1.7%)	(0.0%)
2) The instructor’s feedback was constructive and helpful.	101	58	12	1	1
	(58.4%)	(33.5%)	(6.9%)	(0.6%)	(0.6%)
3) I would have liked to have received more feedback from the instructor.*	8	9	19	80	55
	(4.6%)	(5.2%)	(11.0%)	(46.2%)	(32.2%)
4) I would like to be taught more theory on doctor-patient communication.	4	5	24	64	76
	(2.3%)	(2.9%)	(13.9%)	(37.0%)	(43.9%)
5) I like that the contents of the course were not taught only in a lecture format.	125	33	13	1	1
	(72.3%)	(19.1%)	(7.5%)	(0.6%)	(0.6%)
6) The course concept “role play” of doctor-patient situations is a good idea in general.	107	49	13	3	1
	(61.8%)	(28.3%)	(7.5%)	(1.7%)	(0.6%)
7) It was an important experience to play the role of the doctor or the patient at least once during the course.	62	62	34	11	4
	(35.8%)	(35.8%)	(19.7%)	(6.4%)	(2.3%)
8) The feedback of the other students was constructive and helpful.	98	60	15	0	0
	(56.6%)	(34.7%)	(8.7%)	(0.0%)	(0.0%)
9) I would have liked to have received more feedback from the other students.*	3	18	28	71	51
	(1.8%)	(10.5%)	(16.4%)	(41.5.0%)	(29.8%)
10) The feedback checklists for the observers were helpful.	12	33	63	46	19
	(6.9%)	(19.1%)	(36.4%)	(26.6%)	(11.0%)
11) The learning goals at the end of each lesson were clear to me.	68	65	33	5	2
	(39.3%)	(37.6%)	(19.1%)	(2.9%)	(1.2%)
12) I have gained competence in managing difficult communication situations with patients.	63	79	22	6	3
	(36.4%)	(45.7%)	(12.7%)	(3.5%)	(1.7%)
13) I have learned something useful for my later job as a doctor.	53	80	28	9	2
	(30.8%)	(46.5%)	(16.3%)	(5.2%)	(1.2%)
14) My expectations concerning the course were fulfilled.	71	76	23	3	0
	(41.0%)	(43.9%)	(13.3%)	(1.7%)	(0.0%)

*2 missing values, valid percentages given.

#### Statistical analysis

The analyses were computed with SPSS 18.0. Descriptive statistics were used to describe the sample and the survey results (frequencies, percentages, means, and standard deviations). A two-sample t test and an additional Mann-Whitney U test were used to assess differences between groups (after testing for homogeneity). To account for multiple testing, we applied a Bonferroni correction, which resulted in an adjusted α level of .0295. For the qualitative data, all answers were evaluated, similar contents were categorized, and the frequencies of the categories were assessed. All answers that appeared more than once in the total sample are presented in Table 3.

**Table 3:** Qualitative course evaluation data – content aspects

**Content aspect**	**Statistics**	
	**n**	**%**
	**(frequency of mentioning)**	**(out of N = 182)**
***What did you like about the course?***		
Comfortable/good/relaxed/casual/open/easy atmosphere; no pressure to perform	38	20.9
Constructive/matter-of-fact feedback; constructive cooperation; good collaboration of all participants	24	13.2
Role play instead of frontal teaching; practical use of theory	18	9.9
Discussion; lots of room for discussion; no deadlocked opinion	16	8.8
Good/diversified/interesting/realistic cases	11	6.0
Got to learn about different possibilities/strategies for dealing with different communication situations	6	3.3
***What should be changed about the course and how?***		
Both roles should be played by each student/Everybody should have the chance to be the doctor.	15	8.2
Less theory/fewer models/less repetition of well-known models	10	5.5
Give out patients’ roles earlier/patients should have the chance to prepare better	9	4.9
Conclusion at the end of the lesson unnecessary, redundant information, visualization unnecessary	7	3.8
Do not use feedback sheets	6	3.3
Distribute patient and doctor roles randomly	5	2.7

## Results

### Resources needed for implementation

According to the German Medical Licensure Act (“Approbationsordnung”), the third semester course, which is compulsory for all students, should not exceed a total of 15 participating students. Teaching staff for this course must therefore be provided by the government. The course concept we designed can be implemented in groups with a maximum of 20 students. At the University of Erlangen-Nuremberg, the third semester course is very short as it totals only 12 × 45 minutes. Many universities allow double this amount of time and therefore could easily have time for most of the students to play both roles.

To implement the course concept presented in this paper, the only resource needed is the teacher, and of course a teacher is needed in any other teaching format too. Implementing a new concept always takes additional time and thinking in advance, but this time is not being taken into account as the same applies to any teaching method. Thus, in sum, one could state that our course concept requires departments to provide one staff member per 20 students.

### Evaluation outcome

At the beginning of the course, the vast majority of the medical students (95.6%) thought it was a good idea to be instructed in doctor-patient communication, and more than ¾ (80.8%) preferred interactive methods for gaining competences in this field (see Table 1). Practicing the role of the doctor was rated as more important by the students than playing the role of the patient (79.2% vs. 62.1%). However, only a minority (17.5%) indicated that they would like to take part in a role play more than once and to have the opportunity to try both perspectives. Their reluctance was possibly due to nervousness (at least partly), which was admitted by about ⅔ of the students (64.3%). Almost all students (96.1%) wanted to learn how to communicate in difficult situations with patients, and about half of the students (44.0%) already had experience with patient communication in a professional setting. In conclusion, at the beginning of the course, more than ¾ of the students (76.9%) believed that they would learn something useful in the course for their later professional career in medicine.

**Figure 1:**
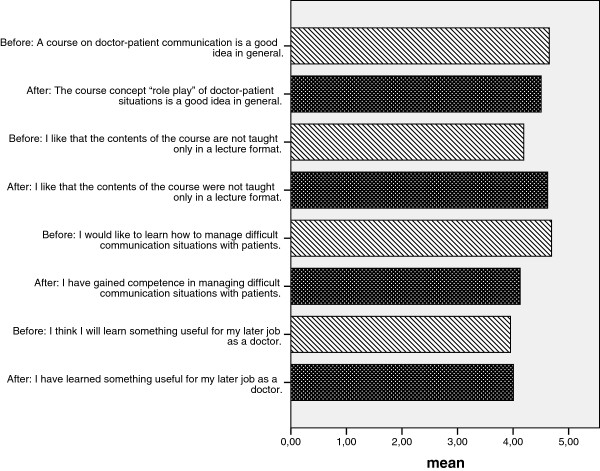
**Changes in items asked before and after the course.** Legend: Attention: For better visibility, the coding of items was reversed: 1 = Do not agree at all, 2 = Hardly agree, 3 = so/so, 4 = agree in part, 5 = Agree completely.

At the end of the course, the majority of the students were satisfied (88.5%) and felt that their expectations of the course had been fulfilled (85.0%) (see Table 2). The feedback after the role plays – from the instructor as well as from other students – was identified as constructive and helpful (91.9%/91.2%) and also sufficient (only a minority wanted more feedback: 10%/12.3%). In comparison with the beginning, 10.6% more (total 91.4%) believed that the interactive method of role playing is more suitable for learning doctor-patient communication than being instructed by a teacher (see Figure 1). Only a minority of 5.2% would have preferred more theory on the topic. Nine out of ten students (90.2%) believed that a course offering the role playing of doctor-patient situations was a good idea in general, and almost ¾ of them viewed the experience of being in the shoes of the doctor/patient as important (71.6%). Whereas, at the beginning, 96.1% wanted to learn how to communicate in difficult situations with patients, a total of 82.1% of all students stated that they actually had learned something about how to manage difficult communication situations (see Figure 1). Furthermore, at the end of the course, ¾ of the students (77.3%) believed that they had learned something useful for their later job as a doctor – almost the exact percentage of students that had this expectation at the beginning of the course (76.9%) (see Figure 1). Concerning the details of teaching, the opinions of the students diverged: Whereas the learning goals of each lesson seemed to be clearly explained and understood (76.9% agreement), the feedback forms that were supposed to facilitate observation and feedback were rated as not so useful (26% rated them as helpful).

Concerning the qualitative data, there were two aspects that appeared with a frequency above 5% concerning things that should be changed about the course and five aspects with a frequency above 5% concerning things that the students liked about the course (Table 3). The most commonly mentioned positive content was the “good/open/relaxed atmosphere” of the course “without pressure” (n = 38; 22.0% of N = 173). The most frequently communicated critique was that “Either both roles should be played by each student or each student should have the chance to play the role of the doctor” (n = 15; 8.7% of N = 173). All categories that were mentioned more than once are presented in Table 3.

We ascertained whether there were significant differences in the evaluation of the course according to the categories (group variables): age, sex, and role played. Most of the differences in the assessment were found for the sex of the student, which resulted in a total of 5 items that were significantly different between women and men. Also, the role chosen by students showed a gender bias: Whereas 62% of the male students chose the doctor’s role, only 40% of the women did so (Chi^2^ test: p = 0.008).

Concerning the age of the students, we ascertained that older students already had more experience with patients before the course and thus gave lower ratings to the competences they gained from the course. They rated the instructor’s feedback as more constructive and helpful (Table 4). Women more than men denied more strongly that there was a need for more theory on doctor-patient communication. Women admitted more often that they were nervous before the role play. Women were also less likely to agree that they preferred more feedback from their fellow students than the male students. In the end, women gave higher ratings than men to the competence they gained in difficult communication situations (Table 4). The students who played a patient’s role more often admitted feeling nervous and confirmed more often wanting to play more than one role than the students who played the doctor’s role. Furthermore, they rated their experience in the role play as less important than the students who played the doctor’s role (Table 4).

**Table 4:** Significant differences in the evaluation of the course according to the role played and age and sex of the students (p < .03 Bonferroni correction; N = 182)

**Item**	**Group**	**Mean (SD)**	**p-value**	**p-value**
			**t test**	**U test**
***Role played***				
I would like to play more than one role.	Doctor	3.26 (1.07)	.011	.008
	Patient	2.70 (1.07)		
When I imagine conducting an interview in front of the class, I get nervous.	Doctor	3.28 (1.14)	.001	.001
	Patient	2.67 (1.16)		
It was an important experience to play the role of the doctor or the patient at least once during the course.	Doctor	1.73 (0.91)	<.001	<.001
	Patient	2.30 (1.05)		
***Age of the student***				
I have already independently conducted patient interviews before (e.g. as a nurse).	<25	2.49 (1.50)	<.001	<.001
	25+	1.47 (1.17)		
The feedback of the instructor was constructive and helpful.	<25	1.57 (0.73)	.003	.033
	25+	1.25 (0.44)		
I have gained competence in managing difficult communication situations with patients.	<25	1.76 (0.75)	.002	.001
	25+	2.54 (1.20)		
***Sex of the student***				
I like that the contents of the course are not only frontally taught.	♂	1.97 (0.91)	.023	.040
	♀	1.69 (0.75)		
When I imagine conducting an interview in front of the class, I get nervous.	♂	3.23 (1.14)	.028	.028
	♀	2.83 (1.22)		
I would like to be taught more theory on doctor-patient communication.	♂	3.90 (1.05)	.001	.002
	♀	4.36 (0.80)		
I would have liked to have received more feedback from the other students.	♂	3.59 (1.02)	.003	.002
	♀	4.06 (0.97)		
I have gained competence in managing difficult communication situations with patients.	♂	2.17 (1.01)	.001	.001
	♀	1.69 (0.73)		

Coding see Tables 1 and 2.

♂ male students ♀ female students.

## Discussion

This paper evaluated the implementation of a practical method for training preclinical medical students in communication skills. Its main goal was to show that practical teaching methods can be implemented without incurring additional costs. This paper not only shows how that can be done but also provides 5 examples of role scripts for different training subjects that can be used by fellow colleagues if they wish to do so. The method that we presented involves a communication training program with peer role plays accompanied by the preparation of a portfolio reflecting two real-patient interviews. Hence, students are first trained in communicational skills in a safe environment. The training is then intensified with real patient encounters during the preparation of the portfolio. In addition, 182 students were assessed with regard to their acceptance of the course concept and their gained competences. To our knowledge, this is the first article (a) to assess a curricular course for preclinical (2^nd^-year) medical students conducted completely as a peer role play course and (b) to publish a concrete training concept that can be implemented by fellow colleagues.

Most studies about role plays or standardized patients in medical teaching concern either additional offers that extend the curricular courses (and therefore do not reach all students) or courses that take place in a later segment of medical education - the “clinical” part of medical studies in Germany. Most of them describe what they did but fail to provide precise steps for how to realize a specific concept [22].

The team at Heidelberg University, for example, provided communication courses for medical students in their final years [11,14,15] to examine differences between different training methods. Many other training classes are for 3^rd^ year medical students [10,16,21], which is the beginning of the “clinical” segment of medical education in Germany.

However, there is some evidence indicating that practical training should be provided as early as possible in medical education: Windish and Eboni [28], for example, recommend that communication skills courses be required early in medical training because communication skills are related to clinical reasoning. Besides being better able to establish a rapport with their patients, students trained in communication made more correct diagnoses than their untrained fellows. In addition to practical reasons, this is another reason why we wanted to establish the course in the preclinical segment of the studies as it may prevent students from forming dysfunctional communication habits that are difficult to “unlearn”. Our data also show that about 40% of the students already had some experience with patient interviews.

A study by Hausberg and colleagues [29] examined an intense training program that included role plays and SPs as well as interviews with real patients for students in the preclinical segment (1^st^ or 2^nd^ year). This program lasted 33 h instead of the 12-h curricular course that is mandatory in Germany. The control group had only the standard course (12 h), which included 3 h of role play. The main part of the intervention group’s course consisted of interviews with psychosomatic clinic patients. After an introductory section with role plays and interviews with standardized patients, every student in the intervention group was given the opportunity to conduct an interview with a real patient. Experts rated video tapes of interviews that were conducted before and after the program by students in both the control and intervention groups. Students in the intervention group performed significantly better than the ones with standard teaching especially in “dealing with emotions”. Taking into account the intense course concept and the long duration of the course (33 h is almost three times longer than our curricular course), the results are not astonishing, but without additional resources, it is nearly impossible to implement a course concept like this into daily teaching.

A second study that examined the effect of communication skills training with “real patients” is a study by Baer and Freer [30] who asked cancer survivors to replay their medical history in interviews with medical students. They too described the use of “real” patients as a very helpful and intense experience for the students. However, this idea was questioned in a study by Thomassen [31], who found significant misalignments between the expectations of patients and doctors-to-be and therefore questioned the “realism” of real patient encounters.

One advantage of our course concept in its current form is that it can be implemented easily without additional staff or time but with a maximum of practical training. One important disadvantage, however, is that students might perform only one role: doctor or patient. This naturally depends on the particular group size. This was the most frequently suggested way to change the course. We also found significant differences regarding the role played in the course: “Doctors” more often rated the experience of playing their role as “important”, whereas “patients” more frequently admitted being nervous before playing their role in front of the class. Afterwards, the “patients” also would have liked the opportunity to play a second role.

Perhaps the most interesting findings of the study are the gender differences. We found that if students were allowed to choose their role on their own, there was a gender bias such that female students more frequently chose to play the role of the patient. This might be due to shyness as female students indicated a significantly higher degree of “nervousness” before role playing in front of the class than male students, and the patient’s role appears to be perceived as the less “dangerous” one. This might also be the reason why women gave higher ratings than men to their acquired competences in communication in difficult situations after the course (overcoming shyness, gaining self-esteem). This result was found in another German study too [21]. In the light of these findings and also taking into account the qualitative data, we therefore suggest that the roles be randomly distributed rather than allowing students to choose. This should prevent any association between “nervousness” and role. However, if students are allowed to choose their roles themselves, female students in particular should be encouraged to play the doctor’s role. Male students, on the other hand, seem to be in need of more theoretical background. One way to take this into account may be to focus more on the theoretical framework in the feedback session – especially with male students. We assume that the important variable is not gender as such but may be the nervousness felt by individual students. Therefore, future studies may wish to focus on different types of helpful feedback in relation to the personality of the student.

The evaluation, especially of the qualitative data, suggests that the course would be most effective if students could play both roles. However, if we did this, we would be able to admit only 10 students per class. Such a small class is not possible at our University due to a lack of resources. Thus, if possible, students should play both roles, but on the other hand, we also wish to sensitize the students to the importance of adopting the patient’s point of view too. Playing the patient’s role additionally seems to result in a higher degree of empathetic abilities in the students [15].

The qualitative data also indicated that the students wanted to be able to prepare for their role play. Thus, not only the “doctors” but also the “patients” should be given enough time to do so – even though this might affect spontaneous communication. Even though a minority of the students indicated that the conclusions and visualizations at the end of the lesson were unnecessary, we suggest keeping them. It is important for the students to receive feedback – not only from the teaching staff but also from their peers. Therefore, the teaching staff must ensure that the students pay attention while watching the role play. There are many claims in the literature that the way feedback is given is very important for students’ acceptance of the course and their learning process [32]. Therefore, we introduced feedback rules in the first session, and the instructors were obliged to pay attention to whether the students complied. More than 90% of the students rated the feedback of their peers and the instructor as constructive and helpful, were satisfied with the course, and indicated that their expectations were fulfilled. Even students who were skeptical before the class admitted after the course that interactive training is a good method for teaching communication skills. Whether or not a feedback sheet like the one we used is necessary remains an open question.

In the future, the first preclinical role-play course as presented in our paper could be followed by a more intense communication skills training course with real patients in the second half of medical studies. Afterwards, in specialty training involving focused training sessions, the young doctors would then be able to hone their communication skills in specific subjects such as palliative care, oncology, and so on. Furthermore, group supervision should be offered so that young doctors can reflect on their experiences with difficult interactions.

One weakness of the study was that the results were assessed only via self-reports. Although one could argue that students might have answered according to social desirability, we could see in the data that students gave differentiated feedback. For example, the majority rated the feedback form as “not so helpful” and gave clear advice on how to improve the course. Furthermore, the effectiveness of role plays in medical training has been demonstrated in a large number of studies and in different countries. Hence, this was not the focus of our study. Most of all, we wanted to create a course concept that can be implemented easily. Nevertheless, studies that compare the effectiveness of different simulation methods and the means of implementation and evaluate long-term effects should be performed in the future.

## Conclusions

The course concept presented in this paper offers a high level of practical communication skills training for preclinical medical students and can be implemented easily in curricular compulsory medical education without incurring additional costs. Students felt the course helped them to prepare for their future careers.

## Competing interests

The authors declare that they have no competing interests.

## Authors’ contributions

KL first introduced the role play course at her own department and developed the role descriptions. She drafted parts of the manuscript, conducted the literature search, developed the structure of the publication, and coordinated the author group. CS was involved in developing the course concept, collected the data from the students, and drafted parts of the manuscript. EG was responsible for the supervision of the course concept and drafted parts of the introduction and discussion in the manuscript. CD was involved in developing the course concept, was responsible for data analysis, and drafted parts of the methods and results sections. All authors have read and approved the final version of the manuscript.

## Pre-publication history

The pre-publication history for this paper can be accessed here:

http://www.biomedcentral.com/1472-6920/14/135/prepub

## Supplementary Material

Additional file 1 Role description sets. Click here for file
