# Supplementary material for: From board to bedside – training the communication competences of medical students with role plays
Source: BMC Med Educ. 2014 Jul 5;14:135. doi: 10.1186/1472-6920-14-135 (PMC4096752; doi:10.1186/1472-6920-14-135)
Supplement: Additional file 1 — Role description sets. [file 1472-6920-14-135-S1.doc]

**Role description sets**

**Session 2 History**

**Patient**

Please imagine as well as you can what it is like to be the person described below.

Your name is Marion Wiegand. You are 25 years old and you took on a post as teacher at a primary school only a few months ago. Now it’s December and both children and colleagues have constantly got colds.

Four weeks ago you unfortunately also started a cold, with a sore throat and coughing occasionally (dry, no sputum). The symptoms varied. At first you still felt okay and didn’t have time to rest because of your work. You are always frantically busy and can’t afford to take sick leave. You hoped that the cold would disappear on its own and simply sucked cough sweets. From time to time you used a nasal spray. Initially the symptoms improved, but they did not go away completely.

For the last 5 days you have been feeling worse and worse. You can’t stop coughing and now you’re coughing up thick, yellowish phlegm. You’ve somehow managed to keep going at work.

Since yesterday evening you’ve been feeling really ill and weak and have a fever of over 39 °C. And to be honest, your ribcage also hurts on the left side when you breathe in and you’re finding it more and more difficult to breathe. You are panting a bit, but are still getting enough air.

You are worried that you might have to be off sick from work for quite a while and have asked your boyfriend to bring you to the physicians’ emergency service.

You are waiting for the general practitioner who’s on duty today. She will start by asking you a lot of questions. Please do not give the information or mention the symptoms that you have until you’re asked to.

You should know the following:

- A year ago you had purulent tonsillitis while you were stressed by doing your exams and had to take penicillin, to which you had an allergic reaction (skin rash).

- You have had no other illnesses and do not take any medications regularly

- You have not been vaccinated against flu (influenza).

**Session 2 History**

**Doctor**

Please imagine as well as you can what it is like to be the person described below.

You are Dr. Ingrid Schulte, a general practitioner and 36 years old. It is Saturday and you are on duty in the physicians’ out-of-hours emergency practice.

It is December and many patients are sitting in waiting rooms with common cold symptoms. Your next patient is Ms Marion Wiegand, 25 years of age, whom you will be seeing for the first time. She is pale and sweaty. Your task is first to find out what symptoms the patient has by asking her specific questions. Look at the illnesses listed below and think what further information you need from the patient in order to obtain a more differentiated picture of the clinical picture. Warn the patient that you will listen to her lung sounds in a minute. The seminar leader will then tell you the examination findings and the diagnosis. Prepare yourself to explain to the patient at the end of the interview how her treatment will continue.

**Background information**

Differential diagnosis

Common cold

- acute viral respiratory infection with cough (only seldom with sputum), rhinitis, sore throat, varying symptoms, fever possible; occurs frequently at the seasonal shift from autumn to winter, duration usually 1 week
- frequently transmitted by infection from persons with viral illnesses of varying types
- Treatment: as a rule 3 days’ rest, increased fluids (tea), keep warm,
- Medication: symptomatic: throat lozenges for sore throat, paracetamol or ibuprofen to bring down the fever.

Influenza

- Symptom onset sudden and acute (patient is still well in the morning, but feels severely ill in the afternoon) with high fever, shivering, feels very ill, headache, aching limbs, dry cough; diarrhoea and abdominal pain possible; duration: as a rule 7-14 days
- Treatment: rest, increased fluids, medications as for common cold

Pneumonia

- acute or chronic inflammation of the pulmonary alveoli with oedema of the pulmonary tissue
- classical bacterial form, feeling of being severely ill, cough with sputum (green, yellow, brownish), chills, fever, difficulty in breathing, possibly thoracic pain on inhaling (pleura affected), usually shallow, laboured and rapid breathing (tachypnoea)
- can occur after a cold that has not been fully cured
- treatment: antibiotics for 7 days, e.g. amoxicillin = a penicillin, in patients with penicillin allergy e.g. clarithromycin = macrolide antibiotic, take antibiotic until used up due to the risk of resistance developing; expectorant (e.g. Mucusolvan syrup), drink a lot, reduce fever higher than 39.5 °C with paracetamol or ibuprofen)
- urgently recommend patient to stay at home in bed, rest very important!
- Give the patient a prescription and a sick note (1 week), after the weekend s/he should see her general practitioner, likely to be unfit for work due to illness for 3 weeks
- patient should return immediately if the symptoms worsen.

**Examination findings given by the seminar leader**

Auscultation of the lungs: left-sided, dorsolateral rales indicating a pneumonia infiltrate in the region where the patient has pain on inhaling, rapid; shallow breathing, but cardiovascular circulation stable.

=> Diagnosis: late complications of a common cold- viral infection, now acute bacterial pneumonia

Outpatient treatment, as cardiovascular circulation stable, no other concomitant diseases

Therapeutic procedure – see pneumonia (antibiotics, sick leave, etc.)

**Session 3 Shared decision making**

**Patient**

Please imagine as well as you can what it is like to be the person described below.

Your name is Mr Walter. You are 72 years old and a widower. You live on a farm in a small village, which you used to run together with your late wife. Now your son is running it together with his wife. You have rarely been ill in your life, have worked hard, but in the last few years you have had difficulty in walking. Your right knee, in particular, becomes painful after you have walked only a short way, for example, when you go to the house next door where your son lives and when you make breakfast for yourself. The pain is particularly bad when you walk upstairs. When you are at home you use a stick to take the weight off your right leg, but unfortunately your left knee is also starting to become painful. When you go to church on Sundays (a walk of about 15 minutes) your knees also swell up. At night or when you are resting you don’t have any pain.

You hesitated to go to the doctor for a long time because your neighbour recently got a new hip joint and was away for 12 weeks. You first went to see your general practitioner who warned you that you would probably have to have an operation. You find the thought of having to go into hospital very frightening. You have never been away from home for longer than a week and want to avoid it at all costs. You simply want something to take away the pain. Your son looks after you so that you don’t have to walk very far and you would be happy if you could simply be fairly free of pain.

You have not yet been prescribed any medication, but when the pain got too bad you took aspirin. That helped sometimes, and sometimes it didn’t.

The doctor you are now seeing is an orthopaedic specialist to whom your general practitioner has referred you. You are seeing him for the first time today.

You walk with a stick, a little bent over, limping. It is mainly your knee that is painful. When you play this role, remember that you are 72!

**Session 3 Shared decision making**

**Doctor**

Please imagine as well as you can what it is like to be the person described below.

Your name is Dr. Meißner and you are an orthopaedic specialist in independent practice. A 72-year-old patient, Mr. Walter, will be coming in to you in a moment. He has been referred by his general practitioner with a diagnosis of suspected osteoarthritis of the right knee joint. You have already done an x-ray which shows a distinct narrowing of the intra-articular space, a typical sign of worn cartilage. Bone spurs (osterophytes) along the edges of the joint sur­faces are also visible.

You take a thorough history to establish what the patient’s actual trouble is. You should con­duct a detailed exploration, including the following questions. What is the patient’s current life situation? When does the pain occur in particular? Does he have pain when at rest?

Your task is to achieve a reliable consensus with the patient after you have taken a thorough history.

Information on osteoarthritis of the knee joint (gonarthrosis):

progressive wear and tear on joints, promoted by, among other things, increasing age and constant, severe strain on the joint with destruction of the cartilage and transformation of the cartilage adjacent to the bone; symptoms: pain, initially on moving, later also when at rest, swelling of the joint, restricted movement.

There are two treatment options for osteoarthritis of the knee.

1. Knee-replacement surgery with an artificial knee:
 (preferably as soon as possible, as long as the patient still has no pain at rest)

Advantages:

- if the operation is successful the patient will probably be pain-free
- the function will probably be maintained

Disadvantages:

- hospital stay
- risks of surgery (haemorrhage, infections, risk of anaesthetic,…) not to be under-estimated, particularly in elderly patients
- risk of inflammation
- approx. 6-12 weeks’ rehabilitation

2. Conservative treatment:

General:

- easing the burden on the joint
- orthopaedic shoes

Physiotherapy (the patient will have to be taken there once a week)

- application of heat and cold
- massages, physiotherapeutic exercises

Medication:

- With this patient you would begin by prescribing ibuprofen as long-term medication. It has an analgesic and anti-inflammatory effect.
- Side effects: gastric ulcer, therefore prophylaxis with proton pump inhibitors (pantoprazol)

Kidney damage – monitoring, blood count, creatinine, particularly during long-term treatment

Locally:

- Ointments (diclofenac)

Advantages of conservative treatment:

- no hospital stay
- no surgery risks (risk of inflammation, anaesthetic …)
- patient can stay at home

Disadvantages of conservative treatment:

- no improvement possible, at best functional stabilisation
- side effects of long-term painkillers (kidneys)

**Session 4 Prevention/Motivation**

**Patient**

Please imagine as well as you can what it is like to be the person described below.

Your name is Alfred Schmidt and you are 56 years old. You like to eat roast pork with dumplings and to drink beer. You also smoke and categorically refuse to do any kind of exercise. You like to watch football and would rather have a barbecue with all your relatives than go for a walk. Your wife has been trying to do sports with you for years and would like you to lose weight. You weigh 105 kg and are 1.75 m tall.

You have been told that your blood pressure is too high and that there’s also some problem with your sugar values. But you can take tablets for the blood pressure and it’ll be ok.

Your general practitioner has often advised you to lose weight, exercise and finally stop smoking. But you didn’t want to listen and simply carried on as before.

Two months ago you had a heart attack. Your wife called the emergency physician. The whole thing really frightened you and your wife. You started to sweat, had an awful, stabbing pain in your chest which radiated into your left arm and it felt like you were dying. At the hospital they implanted a stent. Now everything is totally normal again. You don’t have any symptoms (however, when asked you have to admit that you do rather break out in sweat when you climb the stairs to the first floor and then you sometimes also have a stabbing feeling in your chest.) The doctors at the hospital did tell you that you are a risk patient and that you could have another heart attack any time if you don’t lose weight, don’t do more exercise and don’t stop smoking.

Today you are going to see your general practitioner Dr. Krause for a follow-up check-up. You are going to go even though you really don’t understand what all the fuss is about. You feel well. You’ve long forgotten the fear that you had during the heart attack. You don’t like to think about the fact that your father died of a heart attack.

**Session 4 Prevention/Motivation**

**Doctor**

Please imagine as well as you can what it is like to be the person described below.

Your name is Dr. Elisabeth Krause and you are 45 years old, the mother of 2 children and have been a general practitioner for 15 years. In some cases you treat entire families and know your patients well. You enjoy doing your job, are empathic and try to help each individual anew each time.

Today the 56-year-old Mr Schmidt is coming to see you. He is highly overweight, smokes, likes to drink beer and hates any kind of exercise. His father died of a cardiac infarction. Mr Schmidt has long been seeing you for CHD (coronary heart disease) and hypertension. He is also increasingly developing type 2 diabetes. Two months ago he had a heart attack and was in hospital, where a stent was implanted. You are glad that everything went well. You like him and his wife.

You have often talked to Mr Schmidt himself and his wife about the problems associated with his lifestyle and what you would advise him to do (lose weight, do sports, stop smoking). You have prescribed him medication for the high blood pressure and hope that he takes it regularly.

Today he is coming for a follow-up examination and you want to try together with him to find some basis for making a change. Try to understand why it is so difficult for him and watch for things he says that you can reinforce in the direction of change. What militates FOR behavioural change in the patient and what AGAINST change?

Find out what goals are realistic and implementable for the patient. Come to an agreement with him straight away on appropriate concrete plans for action (e.g. go for a 20-minute walk every day and leave out the cigarette after meals, …).

Background information on cardiac infarction

40% of patients do not survive the first 48h after the infarction; cardiac arrhythmias, atrial fibrillation, ventricular fibrillation and failure of the cardiac pump function are frequent complications.

- late complications: aneurysmas, arrhythmias, cardiac insufficiency, …. another cardiac infarction,
- it is important that the blood pressure is well controlled
- main risk factors for cardiac infarction:

smoking, arterial hypertension, enhanced cholesterol, age, CHD / cardiac infarction in first-degree relatives, diabetes mellitus

Fatty foods, smoking and hypertension lead to deposits in the arteries (atherosclerosis), then CHD, then cardiac infarction.

The deposits can be broken down again if the person eats healthy food, does sports, loses weight and stops smoking.

**Session 6 Breaking bad news**

**Patient**

Please imagine as well as you can what it is like to be the person described below.

You are a 38-year-old woman, Corinna Geissler. You are in a stable relationship and for the last 3 years you have been trying to have a child, without success. Five months ago you finally became pregnant with a girl and since then you have been euphoric, but also always afraid that something might go wrong after all. You had smoked for many years, but stopped immediately when you discovered that you were pregnant (in about the 5^th^ week). Last week you went to your gynaecologist for an ultrasound scan. The scan showed a thickening of the foetus’s nuchal fold and a heart defect (a hole in the wall of the heart). You were told that this is an indication of trisomy 21 and consented to have an amniocentesis. Today you have come back to your gynaecologist who will give you the results of the amniocentesis.

You have spent a terrible week full of worries and fears and you hope against all reason that there has been some error. You are in an emotional turmoil and show that you feel unable to cope with the situation and that don’t know how you will manage to look after a disabled child. Your husband works long hours for a large IT company and is seldom at home. Your parents and parents-in-law also live a long way away.

However, an abortion would be out of the question for you, both ethically and emotionally, since you can already feel the foetus moving. You will be upset if your doctor tries to advise you to have an abortion.

You had imagined that everything would be so lovely and yet actually deep down you had believed that you would not ever have a child. Now you feel guilty for having wanted to have a child so late and believe that the baby’s disability is a kind of punishment – perhaps because you were still smoking. And you are also dreadfully disappointed. You were so happy that you had finally managed to become pregnant – and now this! Never will your child be independent. It will never marry and have its own children. All your girlfriends will look at you strangely. You will never be one of them. You are ashamed of these thoughts and will only tell your doctor about them if you feel well understood.

At the moment everything is too much for you and you can’t concentrate properly. Ask some questions twice or ask about things that your doctor has already explained to you. Actually what you need now is peace and quiet and you want to talk to your husband before you take care of practical things or can take in any more factual information.

**Session 6**  **Breaking bad news**

**Doctor**

Please imagine as well as you can what it is like to be the person described below.

Your name is Dr. Schneider and you are a gynaecologist in a large practice. You have carried out an ultrasound scan on your patient Corinna Geissler, who is 5 months’ pregnant, and found a thickening of the nuchal fold and a heart defect in the baby. This is a strong indication of trisomy 21. You therefore advised your patient to undergo amniocentesis and also carried it out.

The result is clear: Mrs Geißler will in all probability give birth to a baby girl with Down’s syndrome.

You already had the impression at the last appointment that Mrs Geissler is very upset by this prospect and you are still a bit uneasy about the upcoming conversation, in spite of your professional experience, because you cannot gage well how Mrs Geissler is going to react. Please orient your interview techniques towards the SPIKES protocol!

It is possible that Mrs Geissler will have to have a Caesarean section if the foetus cannot be sustained via the umbilical cord for long enough or its heart is too weak. Since it has a heart defect the baby will have to undergo heart surgery after the birth. When exactly that will be cannot only be determined until after it has been born.

Your impression is that Mrs Geissler is very unstable. You cautiously ask about her current situation, what support she has and whether she feels capable of looking after a disabled child. If the mother’s mental health is at risk, termination would still be permitted (the foetus is viable). Find out whether that would be an option for Mrs Geissler.

Establish how much information the patient can handle at this time. You may want to postpone this conversation and give the patient time to think things over.

Information on the symptoms of Down’s syndrome:

- microcephaly

- small nose with a flat bridge of the nose

- epicanthic fold (sickle-shaped skin fold at the inner angle of the eye)

- upward slant of lid axes towards the side

- large tongue and therefore often open mouth

- muscular hypotension: overstretchable limbs, clumsy gait

- developmental disturbances

- heart defects

- diseases of the gastrointestinal tract: duodenal atresia, Hirschsprung’s disease

It is not possible to predict in advance how severe the symptoms will be. Mentally, children with Down’s syndrome are usually happy and loving.

**Session 6 Interview techniques in “difficult situations“**

**Patient**

Please imagine as well as you can what it is like to be the person described below.

Your name is Julia Klose and you are 31 years old. You are studying law. Studying has always been very stressful for you. Your father has a large law office and the plan is that you and your older brother will later take over the office together. Your brother is 33 years old, married with two children and completed his studies as best student in Germany within the standard course duration.

You always found things difficult and would actually rather has been a teacher. You have suffered from stomach pains since doing your school-leaving examination. You have colic-type pain at regular intervals. Sometimes you have diarrhoea and sometimes con­stipation. You have already seen numerous doctors and have also had many colonoscopies done and it simply never ends. You have missed several semesters at university due to the long periods you have spent in hospital. Now you have finally got to the stage of doing your final examination. Your father and brother are waiting impatiently for you to graduate and just at this very moment the pain has become especially bad. So you’re having to go back into the university hospital so that the cause can finally be found and you can get better again. Once again your symptoms improved right after the first few days in hospital. The doctors should hurry up and tell you what you must do when you go home again.

It has never occurred to you that there might be a connection between your symptoms and the university course that you didn’t want to do and other obligations and your difficult family situation. Everyone in your family is very strong, all of them have good genes. All are suc­cessful and you want to be, too, but your mysterious illness gets in your way. You know that it is much better for you to study law than to become a teacher, that was just a crazy idea. Your parents were quite right to force you to do law.

Even if it has not yet been possible to find the right diagnosis you are sure that you are not imagining it. You are not someone who moans or a hypochondriac. You can give as good as you get and you are definitely not crazy. While you have heard that stress can also affect one’s physical condition you are convinced that the actual cause of your illness has simply not yet been found.

**Session 6 Interview techniques in “difficult situations”**

**Doctor**

Please imagine as well as you can what it is like to be the person described below.

You are Dr. Neuner, a resident physician/intern working in a surgical department. The 31-year-old patient has been here before, a year ago, when she was in the care of a colleague. A thorough diagnostic work-up was done at the time and now another one has been carried out. You don’t know the patient, but are to discuss the results with her today. The x-ray, CT, MRI scan and blood count show no pathological findings. The gastroscopy and colonoscopy were also without pathological findings. Physically the patient is perfectly healthy and in her file it also says that her symptoms always suddenly improve when she is in hospital.

You don’t want simply to send the patient away. You are of the opinion that it will not help to do further diagnostic tests and examinations and that patient should urgently be referred to the Psychosomatic Clinic before her symptoms get worse or continue for several years. You believe that there must be another, non-physical origin of her symptoms.

You talk to the patient very empathically and try to steer the conversation in a direction that allows her to recognise a connection between some details of her life and her symptoms. Ask about her studies at university and her motivation, whether that is what she always wanted to do and whether she finds her studies easy. Ask her also about her siblings and parents.

You want cautiously to explain to the patient that a physical cause of her symptoms has been ruled out and that you would like to refer her to the Psychosomatic Clinic. Emphasise that the psyche needs to be cared for just like the body and that stress –including mental stress – can definitely have an effect on the body.
